# Policy Resistance Undermines Superspreader Vaccination Strategies for Influenza

**DOI:** 10.1371/journal.pcbi.1002945

**Published:** 2013-03-07

**Authors:** Chad R. Wells, Eili Y. Klein, Chris T. Bauch

**Affiliations:** 1 Department of Mathematics and Statistics, University of Guelph, Guelph, Ontario, Canada; 2 Center for Advanced Modeling, Department of Emergency Medicine, Johns Hopkins University, Baltimore, Maryland, United States of America; Pennsylvania State University, United States of America

## Abstract

Theoretical models of infection spread on networks predict that targeting vaccination at individuals with a very large number of contacts (superspreaders) can reduce infection incidence by a significant margin. These models generally assume that superspreaders will always agree to be vaccinated. Hence, they cannot capture unintended consequences such as policy resistance, where the behavioral response induced by a new vaccine policy tends to reduce the expected benefits of the policy. Here, we couple a model of influenza transmission on an empirically-based contact network with a psychologically structured model of influenza vaccinating behavior, where individual vaccinating decisions depend on social learning and past experiences of perceived infections, vaccine complications and vaccine failures. We find that policy resistance almost completely undermines the effectiveness of superspreader strategies: the most commonly explored approaches that target a randomly chosen neighbor of an individual, or that preferentially choose neighbors with many contacts, provide at best a 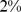 relative improvement over their non-targeted counterpart as compared to 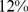 when behavioral feedbacks are ignored. Increased vaccine coverage in super spreaders is offset by decreased coverage in non-superspreaders, and superspreaders also have a higher rate of perceived vaccine failures on account of being infected more often. Including incentives for vaccination provides modest improvements in outcomes. We conclude that the design of influenza vaccine strategies involving widespread incentive use and/or targeting of superspreaders should account for policy resistance, and mitigate it whenever possible.

## Author Summary

Superspreaders are the small number of individuals responsible for the majority of infections. Theoretical models have shown how vaccinating superspreaders can be a highly efficient way to control disease. However, these models neglect behavior by assuming that superspreaders will always agree to be vaccinated. This is a problematic assumption for influenza vaccination, which is voluntary in most populations, and for which vaccine coverage is often suboptimal. We developed a model of seasonal influenza transmission on a network of individuals who make decisions about whether or not to get vaccinated based on known determinants of vaccine uptake, such as personal infection history, perceived vaccine risks, and social influences. We found that, because of feedbacks between disease spread and individual vaccinating behavior, attempts to boost vaccine coverage in superspreaders through the use of incentives or recruiting by social contacts are almost completely undermined by such feedbacks. For example, higher vaccine uptake in superspreaders reduces influenza incidence, which in the next season reduces the perceived need for vaccination among non-superspreaders, who then do not become vaccinated as much. Our results suggest that the design of potential strategies to reach influenza superspreaders should account for behavioral feedbacks, since they may blunt policy effectiveness.

## Introduction

Seasonal influenza imposes a significant health burden: in the United States alone there are an estimated 25–50 million cases per year, with 30,000 deaths and numerous hospitalizations, especially among the elderly and individuals with severe medical conditions [1], [2]. Vaccination generally commences in September prior to the influenza season and is non-mandatory for the general public [1]–[4]. The question of whether to focus efforts on increasing vaccine coverage in children (who spread a disproportionate amount of infection) or the elderly (who suffer the greatest health burden from infection) has received significant attention in the transmission modelling literature [5]–[7]. Much of this work indicates that immunizing children might be a more effective way to reduce overall disease burden in the population. However, vaccine coverage has not significantly expanded in children. This leaves room for considering alternative strategies.

Many infectious diseases exhibit a highly heterogeneous form of transmission known as “superspreading”, wherein a minority of individuals are responsible for the majority of secondary infections [8]–[12], and it is possible that influenza also exhibits this property [13]. In a contact network, a superspreader can be represented as an individual with a very large number of contacts. Network-based infectious disease transmission models show how targeting superspreaders can be a highly effective (and efficient) form of infection control [14]–[20]. This suggests there may be value in exploring the possibility of immunizing influenza superspreaders, hence the focus of our analysis in this paper.

Transmission models generally treat vaccine coverage as a fixed control parameter [21], requiring the implicit assumption that desired vaccine coverage can always be achieved. However, public health authorities do not decide influenza vaccine coverage because they do not control individual vaccinating decisions. Instead, they control decisions such as where to set up immunization clinics, how to disseminate information, and whether to offer incentives to get vaccinated. Using a theoretical model to address factors that public health actually controls requires incorporating individual vaccinating behavior into the model. However, models of superspreader vaccination strategies usually assume that targeted individuals will always agree to be vaccinated to an arbitrarily specified level of vaccine uptake [14]–[16], [18].

Incorporating behavior into transmission models is increasingly important in an age of vaccine exemption, especially for influenza vaccine, for which coverage is typically suboptimal [22], [23]. Combining incentive use with targeting of influenza superspreaders could potentially be very effective, but behavioral feedbacks need to be considered in program design.

Previous research has integrated behavioral modelling with transmission modelling to explore aspects of vaccinating behavior for various vaccine-preventable infections (see Refs. [24]–[26] for reviews). Earlier approaches used compartmental models, but more recently, researchers are also using simulation models where transmission of infection and/or information occurs through social contact networks [27]–[31]. Transmission patterns can change significantly–even qualitatively–when transmission occurs through a network rather than in a homogeneously mixing population, and vaccinating behavior can change accordingly. The proliferation of recent papers considering vaccinating behavior on social contact networks prevents our providing a comprehensive survey here. However, to our knowledge, these previous models have not analyzed how behavioral feedbacks influence the effectiveness of vaccination strategies that target superspreaders, nor have most of them explored how incentive use influences vaccination behavior.

Here, we analyze an agent-based simulation model that couples seasonal influenza transmission on an empirically-based contact network with a psychologically realistic model of individual vaccinating decisions. We explore the effectiveness of incentive programs and targeted superspreader vaccination strategies. Our objectives are to understand: 1) whether superspreader vaccination strategies remain effective when behavior is accounted for; 2) whether economic incentives improve the effectiveness of such strategies; and 3) how perceived vaccine efficacy and the resulting vaccinating decisions are determined by interactions between network structure, transmission heterogeneity, and vaccine-disease dynamics.

## Model

### Population structure

For our baseline analysis we generated ten contact networks of 10,000 nodes each, by sampling subnetworks from a large contact network derived from empirical contact patterns in Portland, Oregon [32]–[34]. We ensured that the resulting node degree distribution and clustering coefficient matched that of the full empirical network (see Text S1). For influenza, susceptible individuals are recruited primarily through immunity waning, hence we assumed that the networks remained static, with no immigration or emigration. In our sensitivity analysis we explored hypothetical networks with exponential and Poisson node degree distributions.

The contact network contains individuals representing the full spectrum of neighborhood sizes and does not impose a dichotomy between superspreaders and others. However, to assist with interpreting the output of our simulations, we defined a superspreader as an individual who infected more than the 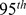 percentile from a Poisson distribution with mean 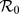, where 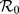 is the basic reproduction number for the “null” deterministic model's approximation. Approximately 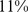 of individuals in the empirically-based network met this definition of superspreaders (see Text S1 for details) [11].

### Disease dynamics

We assumed a Susceptible - Infected - Recovered - Vaccinated - Susceptible (*SIRVS*) natural history. An infectious individual transmits influenza to a susceptible contact 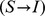 with probability 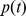 per day, where 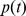 varies seasonally. An infectious individual moves to the recovered state 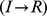 after a number of days sampled from a Poisson distribution with mean 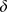 days. A recovered individual becomes susceptible 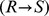 with probability 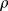 per season (natural waning immunity). A vaccinated individual becomes susceptible 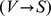 with probability 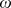 per season (vaccine waning immunity). Vaccination has no impact on individuals who are in the naturally immune 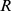 state and the vaccine efficacy is 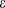. Symptomatic infection occurs with probability 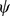. In our sensitivity analysis, we also allowed for heterogeneity with respect to the infectious period 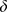 and the infectivity 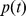. This creates additional sources of heterogeneity that may cause some individuals to become superspreaders. More details appear in Table 1, Table S1 and Text S1.

**Table 1:** The values and descriptions of the parameters used in the simulations.

Parameter	Description	Value	Reference
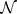	Number of individuals in network	10000	assumption
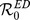	Null Deterministic Basic Reproductive Value (empirically-based)	3.45	calibrated **; [40], [53]–[55]
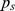	Change in Seasonality Amplitude (empirically-based)	0.03	[53], [56]
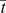	Shift in Seasonality function (empirically-based)	120	calibrated**
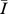	Number of Exogenous Infections (empirically-based)	11	calibrated**
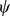	Probability of influenza being symptomatic	0.70	[57]
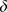	Average number of days to move from state 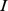 to state 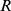 (recovery rate)	5	[44], [57]–[59]
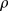	Probability of moving from state 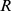 to state 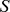 , per season	0.25	[58], [60], [61]
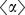	Average incidence for niILI, per day	0.00035†	[62]
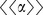	Variance of incidence for niILI	12.25 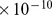	calibrated††
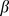	Probability of an individual mistaking niILI for influenza	0.50	assumption
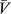	Number of individual's contacted for vaccination, per day	20	assumption
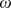	Probability of moving from state 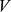 to state 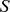 , per season	0.50	[63]
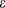	Vaccine efficacy	0.70	[41], [64]
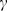	Probability of experiencing vaccine complications, per vaccination	0.01	[45]
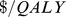	Cost per Quality Adjusted Life Years	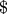 50,000	[43]
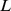	Baseline payoff	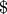 50,000	assumed
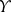	Monetary value of the incentive	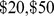	[41], [44]–[46], [49], [50], [65]
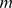	Memory decay rate, per season	0.30	calibrated
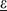	Minimum perceived vaccine efficacy	0.65	calibrated
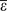	Maximum perceived vaccine efficacy	0.90	assumption
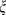	Vaccine efficacy memory decay rate factor	15	assumption
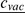	Minimum cost of vaccination	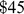	◊ [41], [44]–[46]
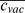	Additional cost of vaccination due to a complication	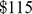	assumption [41], [44]–[46]
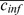	Maximum cost of infection	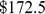	[43]
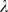	Weight assigned to personal experiences	0.50	assumption
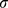	Probability that the individual imitates	0.50	assumption
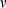	Strength of preference to imitate contacts	0.50	assumption
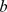	Parameter for vaccine uptake equation (empirically-based)	3950	calibrated

The values were calibrated for each network using the passive vaccination approach.

* The values 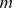, 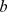 and 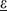 were calibrated such that the average annual vaccine coverage on each network was approximately 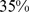 using appropriate values.

**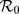 was used in calibrating influenza incidence (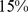) using values similar to influenza's 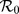 [40]–[42] on each network such that the average peak of prevalence occurred between January 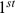 and February 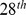 [66].

† The value for 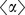 was calculated such that the annual incidence of non-influenzal influenza-like-illness (niILI) was 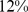, corresponding to the ratio of niILI incidence to influenza incidence estimated in [62] and multiplied by the average annual influenza incidence (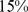) [37], [40]–[42].

†† The variance for 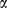 was calibrated such that the log-normal distribution best resembled the shape of a normal distribution.

◊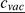 was computed as the cost of the actual vaccination 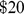 and plus the time required to receive the vaccination 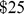.

### Vaccinating behavior

We structured the vaccination decision-making submodel according to known determinants of influenza vaccine acceptance. Empirical studies have identified that perceived vaccine effectiveness, previous acceptance of vaccine, past experiences with infection and vaccine complications, social influence, and perceived susceptibility are correlates of vaccine acceptance [35], [36]. Although the data in these studies are not detailed enough to favor particular functional forms governing these effects, it is nonetheless possible to construct functional forms that are qualitatively consistent with them, which is also the approach adopted in some other models [37].

The payoffs for strategy choices are given by
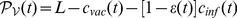
(1)

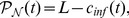
(2)where 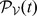 is the payoff to vaccinate for season 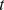, 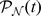 is the payoff not to vaccinate, 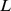 is the baseline payoff (a state of perfect health), 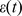 is the perceived vaccine efficacy, 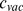 is the cost of vaccinating and 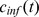 is the perceived infection cost [37].

The perceived infection cost 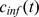 incorporates perceived susceptibility and past infection experiences. Perceived susceptibility is expressed through past influenza incidence in the population. Past infection experience is expressed through the time since the individual's last perceived infection, 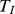. Hence
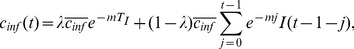
(3)where 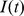 is the influenza incidence in season 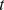, 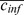 is the penalty for being infected, 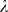 controls the relative importance of personal history versus population history, and 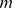 is the memory decay rate. Severe outcomes are implicitly accounted for in 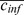, which represents the combined foreseen risk of infection and any resulting complications. Thus, this equation captures how individuals use past experiences to guide future vaccinating decisions.

The perceived vaccine efficacy 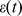 for an individual in season 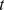 generally differs from actual efficacy 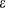 and is given by
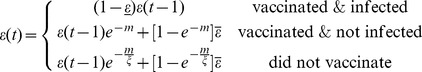
(4)where 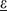 controls how quickly perceived vaccine efficacy drops upon a perceived vaccine failure, 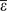 is the maximum perceived vaccine efficacy, and 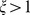 is a decay factor which causes memory of a previously ineffective vaccination to fade at a slower rate 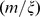 than a successful vaccination, since they have less information with which to update their impression [37]. The asymmetry between an event where individuals vaccinate and become infected versus an event where they did not vaccinate arises because of the distinction between “evidence of absence” and “absence of evidence”. Only symptomatically infected individuals update their values of 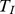 and 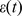.

The cost of vaccination also incorporates past experience:
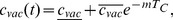
(5)where 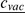 represents time and economic costs, 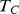 is the time since the last perceived vaccine complication, and 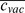 is the perceived cost of a vaccine complication. The probability an individual perceived a complication upon vaccinating is 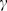.

We incorporate social influence through a learning process. Empirical studies of determinants of vaccine acceptance suggest that individuals form opinions through communicating with their peers and sharing personal experiences [35], [36]. Hence, before each vaccination season, an individual engages in a social learning process with probability 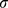, sampling another individual at random and replacing their 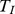, 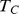 and 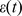 with the average of their pre-existing value and that of the sampled individual, weighted by 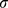 and 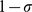 respectively. This captures both the tendency to personalize the experiences of others, as well as habit, since strategies change more slowly as 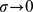. This mechanism of social learning is similar to that used in a previous behavior-incidence model that was validated against empirical data [38].

We also account for the impact of non-influenzal influenza-like-illness (niILI) on decision making, since niILI can be mistaken for true influenza and thus alter 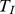 and 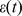. The probability an individual experiences niILI each day is 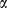, where 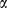 is sampled from a log-normal distribution parameterized from empirical data on niILI incidence. An individual mistakes niILI for true influenza with probability 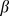, in which case 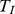 and 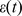 are updated accordingly (see Text S1 for details).

### Vaccination strategies

Passive Vaccination (PV) is the baseline strategy corresponding to how most influenza vaccination programs are designed: vaccines are made available (e.g. at drug stores, public health clinics, doctors' offices), opening times are widely disseminated, and individuals seek out vaccination on their own, without being individually recruited by public health.

To capture this, we assume an individual decides to get vaccinated for the current season with probability 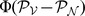, which is a sigmoidal function of 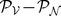 such that 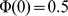, 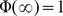, and 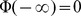 (see Text S1). We assume individuals can be vaccinated only between September 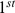 (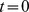) and December 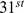 [4]. Those who choose to vaccinate have their times of vaccination distributed throughout this period according to a process described in Text S1. If an individual perceives having been infected by influenza before it is their time to become vaccinated, they do not seek vaccination.

In addition to making PV available, public health may also implement one of four pro-active strategies: 1) random vaccination (RV) which targets a randomly chosen individual; 2) nearest neighbor vaccination (NN), which targets a randomly chosen individual and one of their neighbors (i.e. contacts) 3) chain vaccination (CV), which either targets a randomly chosen individual or a neighbor of an individual targeted the previous day; and 4) improved nearest neighbor vaccination (INN) which targets a randomly chosen individual and one of their most popular neighbors. Under INN, “popular” means having the highest degree, and we assume imperfect knowledge of a neighbor's neighborhood size. We refer to NN, CV and INN as superspreader strategies, as their objective is to target individuals with a large number of contacts [15], [16], [18], [39].

The number of individuals targeted by public health each day is held constant at 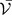 for all strategies. In each case, if the targeted individual did not already decide to vaccinate under PV, they reconsider: they undergo the social learning process again and agree to be recruited for vaccination with probability 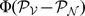. In our sensitivity analysis we also ran simulations where each targeted person was automatically recruited, corresponding to a situation where behavior is neglected (NB). More details on the pro-active strategies appear in Text S1.

### Incentives

We allowed for the use of economic vaccination incentives under the pro-active strategies. Each time an individual is targeted they receive an incentive of value 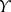 if they get vaccinated during the current season. An individual can receive multiple incentives. Hence superspreaders should receive more incentives, since they are likely to be targeted multiple times under NN, CV and INN. We considered 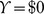 (baseline), 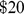, and 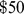. With incentives, the probability of vaccinating becomes a function of 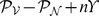 (where 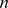 is the number of times they have been targeted) instead of 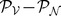. In order to express 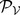, 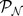 and 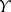 in the same payoff currency, 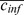, 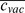, and 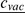 were expressed in quality-adjusted life years (QALYs) (see Text S1).

### Model calibration and simulation design

For each of the ten networks, the transmission probability and amplitude of seasonality were calibrated so that the average seasonal incidence of influenza in the absence of vaccination was 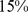 [40]–[42], and prevalence peaked between January and February. 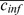 was based upon utility scores derived from patient surveys [43]. 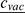 was based on published vaccine costs [41], [44]–[46]. 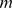 and 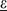 were calibrated such that the average annual vaccine coverage was 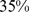. For a 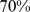 efficacious vaccine, vaccine coverage of 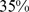 reduces seasonal influenza incidence by about 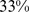 (Table 2), in line with what is expected for an imperfect vaccine covering one-third of the population. Examples of calibrated time series of annual coverage and weekly incidence appear in Figure S1. For each network we generated 400 realizations of 150 years each, discarding the first 125 years to avoid transient effects.

**Table 2:** Average influenza incidence 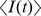 and vaccine coverage 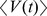 in the entire population, and just in superspreaders (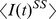, 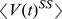).

Strategy	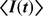	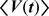	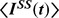	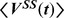
No Vaccination	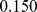	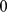	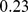	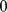
PV	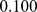	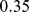	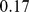	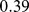
PV+RV	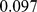	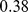	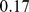	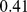
PV+NN	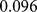	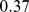	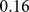	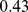
PV+CV	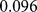	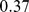	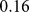	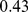
PV+INN	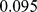	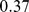	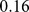	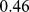
PV (NB)	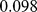	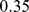	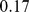	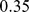
PV+RV (NB)	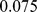	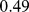	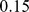	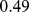
PV+NN (NB)	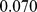	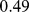	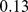	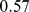
PV+CV (NB)	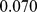	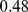	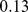	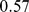
PV+INN (NB)	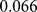	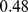	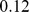	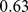
PV+RV 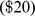	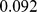	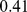	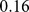	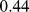
PV+NN 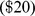	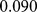	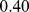	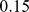	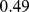
PV+CV 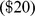	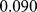	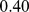	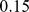	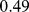
PV+INN 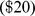	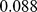	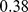	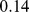	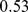
PV+RV 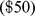	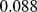	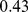	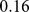	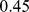
PV+NN 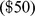	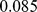	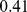	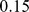	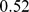
PV+CV 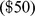	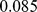	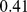	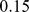	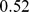
PV+INN 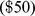	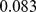	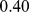	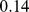	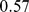

Numbers represent mean of 400 simulations (standard deviations were very small). NB indicates that vaccinating behavior is ignored, 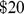 (respectively 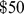) indicates that 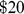 (respectively 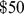) incentives are used. The strategies listed without any parentheses (rows 2-6) pertain to the baseline model: strategies with behavior but no incentives.

## Results

In the absence of incentives, the improved nearest neighbor strategy (INN) is the most effective in reducing influenza incidence, followed by chain vaccination (CV), nearest neighbor (NN), random vaccination (RV), and the baseline strategy of passive vaccination alone (PV) (Table 2).

This relative ordering is to be expected, since previous research shows the advantages of targeting individuals with many contacts [14], [16]–[20]. However, feedbacks due to the dependence of vaccinating decisions on infection history generates some surprises [26], [38], [47], [48]. In this system, the feedbacks manifest as policy resistance [48], where the response of the population to an intervention (in this case, pro-active strategies and incentives) tends to reduce the effectiveness of the intervention. In our model simulations, policy resistance arises because increased vaccine coverage in one season reduces incidence due to both direct and indirect (herd) protection, which in turn disincentives vaccination in future seasons, since decisions are based partly on infection history and perceived vaccine failure/complications. An additional source of policy resistance in this system is the tendency for pro-active strategies to waste recruitments on individuals who already decided to get vaccinated under passive vaccination, or who have already been infected (this is a problem especially among superspreaders, who are both targeted more often and tend to get infected earlier in the season) (Table S2). On average, only 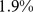 of the population was vaccinated through being contacted through a nearest neighbor under INN; whereas under NN and CV the percentage was 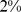.

**Figure 1:**
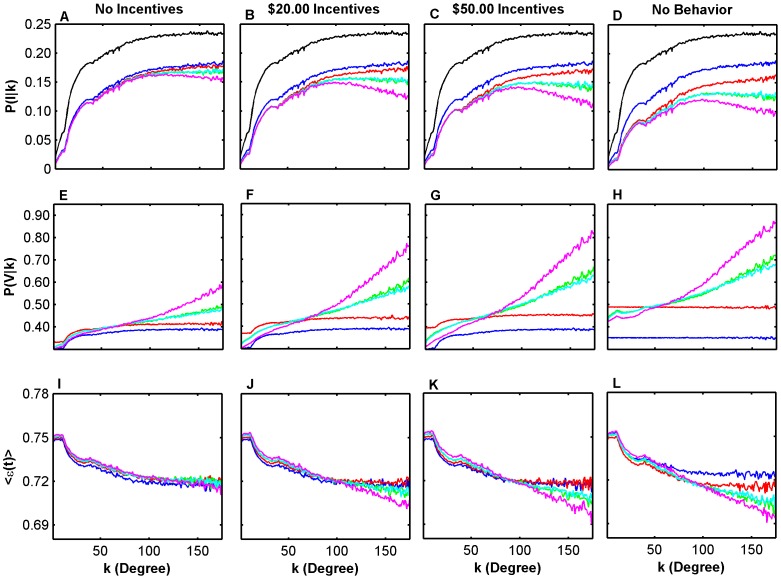
Model outcomes as a function of neighborhood size 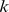. Average probability of being infected (a–d), probability of being vaccinated (e–h), and perceived vaccine efficacy (i–l) for the scenarios of no incentives (a, e, i); 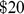 incentives (b, f, j), 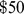 incentives (c, g, k), and no behavior (d, h, l). Strategies include no vaccination (black), passive vaccination (blue), random vaccination (red), nearest neighbor vaccination (green), chain vaccination (light blue), and improved nearest neighbor vaccination (purple).

Policy resistance almost completely undermines the benefits of using pro-active strategies: passive vaccination (PV) reduces seasonal influenza incidence from 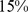 to 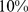, but implementing improved nearest neighbour vaccination (INN) on top of that provides only slight additional reductions, down to 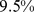. The other pro-active strategies (NN, CV, RV) are even less effective, reducing incidence to 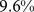 or 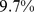 (Table 2). Moreover, among pro-active strategies, superspreader strategies are only marginally more effective than random vaccination (RV) (Table 2). As expected, the superspreader strategies improve vaccine coverage among superspreaders. However, this is offset by lower coverage among non-superspreaders. As a result, the average vaccine coverage under superspreader strategies is the same as under random vaccination (Figure 1, Table 2).

The impact of policy resistance is made clear by considering the case where vaccinating behavior is neglected (by assuming that targeted individuals are automatically recruited for vaccination). Neglecting behavior (NB) significantly overestimates both effectiveness and vaccine coverage for the pro-active strategies, both in superspreaders and non-superspreaders (Table 2). Hence, without accounting for behavior, we might have concluded that superspreader vaccination strategies can be significantly more effective than their non-targeted counterpart, but if we take behavior into account, their impact is greatly diminished.

We note that the slightly higher effectiveness of the improved nearest neighbor strategy also arises because by preferentially immunizing those individuals with a large number of contacts, susceptible individuals tend to be clustered together on the network, reducing the opportunities for the susceptible-infected contacts necessary for transmission (Table S3).

Individuals with more neighbors were more likely to be infected (Figure 1a)–d)). This resulted in a higher probability of them getting vaccinated (Figure 1e)–h)), but it also caused them to perceive the vaccine to be less effective (Figure 1i)–l)), on account of higher infection rates causing higher rates of perceived vaccine failure.

The effect of adding vaccinating incentives is likewise blunted by policy resistance (Table 2). Any increase in vaccine coverage due to use of incentives reduces incidence, which in turn disincentivizes future vaccine uptake (especially among superspreaders under passive vaccination, Table S4). Also, incentives often reach individuals who are already prone to get vaccinated (Table S2). However, modest improvements in program effectiveness due to the use of incentives are still possible. For example, adding a 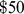 incentive to the improved nearest neighbor strategy reduces influenza incidence from 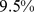 to 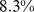 (Table 2).

We estimated the net per capita costs (total vaccine costs plus total infection treatment costs per member of the population) for each strategy. The least expensive strategy was the improved nearest neighbor strategy (INN) without incentives, at a cost of 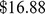 per capita. In contrast, passive vaccination on its own (PV) costs 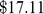 per individual because infection costs are higher under PV than INN. These results assume the administrative costs of vaccination are the same for passive versus pro-active strategies, although in reality the marginal cost per vaccinated person may be higher under pro-active strategies, especially if they involve targeting superspreaders. We ran additional simulations where there was an additional marginal cost for recruiting contacts (as under NN, CV and INN), finding that the marginal cost for recruiting contacts under INN would have to be at least 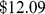 per recruited individual before INN becomes more costly than PV.

Using vaccinating incentives increased the total cost of all strategies, but not always significantly. Further details appear in Text S1 and Table S5.

On average, most individuals received few incentives and only a few individuals received many incentives (Figure S2). Superspreaders tended to receive more incentives by virtue of having more contacts, but the benefit of this was partly mitigated by the fact that they are likely to be infected and/or seek vaccination earlier in the season than individuals with few contacts, and hence have less time to accumulate incentives.

Our baseline assumption was that superspreading is driven only by heterogeneity in neighborhood size (node degree). Incorporating heterogeneity into the infectious period, transmission rate, or both did not significantly impact the results (the superspreader strategies become slightly less effective in the absence of incentives). We suspect these forms of heterogeneity did not make a difference because an individual's infectious period and infectiousness were not correlated to their node degree, meaning that superspreader strategies on average do not target individuals with higher infectiousness or longer infectious period. This causes differences in effectiveness between the various strategies to be averaged out. Were correlations to exist between node degree on the one hand, and infectious period or transmission rate on the other hand, then we speculate the results could change qualitatively, either in the direction of greater effectiveness of superspreader strategies, or lesser effectiveness, depending on whether the correlation was positive or negative, respectively.

For simulations on the hypothetical Poisson or exponential networks instead of the empirically-based network, we found that the pro-active strategies continued to provide very small reductions in incidence compared to passive vaccination alone (Table S6 and Table S7). Results were also qualitatively unchanged when incentives were distributed only to the recruited neighbor of individuals targeted under NN and INN (Tables S2, S5); however, the cost of the policies was reduced due to fewer incentives being distributed (Table S5).

## Discussion

Previous models of superspreader vaccination strategies have shown how targeting individuals with a very large number of contacts can be a very effective way to control infection [14]–[20], [39]. These models have generally assumed that targeted individuals will always agree to be vaccinated. For voluntary influenza vaccination, this assumption may introduce inaccuracies, since individual choice is a major determinant of influenza vaccine uptake [22], [35], [36].

Here, we developed a psychologically structured model of influenza vaccinating behavior and coupled it to a model of seasonal influenza transmission through an empirically-based contact network. Our assumptions about vaccinating behavior were based on empirical studies exploring determinants of vaccine uptake [35], [36]. We found that three of the most commonly investigated superspreader vaccination strategies (nearest neighbor, chain vaccination, and improved nearest neighbor) provided little or no improvements over their non-targeted counterpart (random vaccination).

This surprisingly strong policy resistance is driven by multiple mechanisms: individuals are less likely to get vaccinated if their most recent influenza infection was a long time ago, if they perceive low susceptibility to infection (which can emerge from herd immunity generated by vaccination), or if they perceive recent vaccine complications or low vaccine efficacy. The presence of non-influenzal influenza-like illness (niILI) reinforces this because it can create the perception of vaccine failure. Moreover, superspreader strategies tend to reach individuals who are already more prone to get vaccinated without the need for active recruitment on account of their history of more frequent infections. Contact-based recruiting was also stymied by the fact that neighbors tended to share similar experiences and information, which led to neighbors more often than not sharing the same opinion regarding vaccination [27], [37].

Providing vaccination incentives boosted effectiveness somewhat, including for superspreader strategies, although these gains were again partly mitigated by policy resistance. The most effective overall strategy was the improved nearest neighbor strategy (INN) with 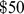 incentive: this strategy reduced the average annual influenza incidence to 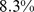 (compared to 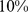 under passive vaccination). The improved nearest neighbor strategy may also be cost-saving compared to passive vaccination. A few empirical studies have found that incentives can increase vaccine uptake [49], [50]. However, these studies focused on incentivization for small groups of elderly individuals over the course of a single season, not widespread incentivization for individuals with a large number of contacts.

Individuals with more contacts were more likely to be infected, which agrees with results from past models [51], [52]. This resulted in an individual's perceived vaccine efficacy declining with their number of contacts. This is a potential barrier in superspreader vaccination compliance. More generally, how perceived vaccine efficacy evolves over time and in response to disease dynamics and the collective effects of individual vaccinating decisions merits further study.

As with any model, our model made simplifying assumptions. For example, we assumed a targeted individual who is asked to recommend a contact for vaccination would always comply. We could address this limitation by introducing a parameter for compliance failure. This would result in a similar strategy to the chain vaccination strategy, where occasionally the recruitment process jumps to another individual rather than continuing along the chain of contacts. We also neglected age structure in the model, which did not allow us to address issues such as age-related heterogeneity in infection severity, and correlations between infection severity and neighborhood size.

Incorporating greater heterogeneity into the model by stratifying individuals with respect to age would increase model realism. It would also allow us to address other objectives such as how incentives can be designed to boost vaccine coverage in children. However, this would also increase model complexity, and given that current model already required dealing with the extra complexity of incorporating behavior, we opted for the incremental approach of first developing a model without age structure. Another aspect of model development that requires greater attention is the functional forms used to capture psychological effects such as the role of past experiences and social influences, since typically more than one functional form is qualitatively consistent with existing data on determinants of vaccine uptake.

These areas suggest potential for further work at the interface of theoretical modeling and empirical surveys. In particular, surveys of determinants of vaccination behavior can be designed to better meet the needs of models that couple disease dynamic models to vaccinating behavior models, for instance by helping to determine which functional forms best capture psychological effects. This will require collecting new psychological data from study populations. In other cases, these models suggest predictions which can be tested. For example, our model predicted that superspreaders would perceive a slightly lower vaccine efficacy than non-superspreaders, and it would be interesting to see whether this effect holds true for any populations.

Thoroughly validated “behavior-incidence” models of influenza vaccinating behavior will help public health authorities to optimize influenza vaccine programs. However, as we have found here, vaccination strategies that target superspreaders and/or provide vaccination incentives must be carefully designed to mitigate the potentially strong effects of behavioral feedbacks and policy resistance.

## Supporting Information

Figure S1The top row, a)–c), shows the degree distribution for each network (black) compared to the original empirical network (gray) of Portland, Oregon [32]–[34]. The second row, d)–f), clearly shows that prevalence peaks between the beginning of January and the end of February. The third row, g)–i), shows the prevalence over many years under no vaccination and the fourth row, j)–l), shows the average vaccine coverage over the years under passive vaccination. The average time for peak of prevalence: Empirically-based 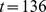, Exponential 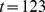 and Poisson 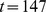. The approximate average duration of the season: Empirically-based 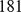 days, Exponential 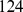 days and Poisson 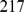 days.(TIFF)

Figure S2 A semi-log plot of the probability of number of incentives received and used by superspreaders (solid line) and non-superspreaders (dashed line) for the four different vaccine strategies a) random vaccination b) nearest neighbor vaccination c) chain vaccination and d) improved nearest neighbor vaccination.
(TIFF)

Table S1The values and descriptions of the parameters used in the simulations for the Poisson and exponential networks. The values were calibrated for each network using the passive vaccination approach. 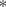 The values 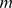, 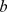 and 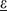 were calibrated such that the average annual vaccine coverage on each network was approximately 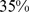 using appropriate values; the monthly vaccine uptake was based on the 2010–2011 influenza season [67]. 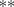 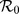 was used in calibrating influenza incidence (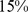) using values similar to influenza's 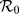 [40]–[42] on each network such that the average peak of prevalence occurred between January 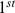 and February 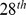 [66].(PDF)

Table S2Percentage of recruitments where recruitment did not matter, whether due to infection, already vaccinated or will be vaccinating in the future (Useless) and the percentage of incentives actually used (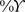 Used). NB indicates the scenario where vaccination behavior is entirely ignored, 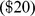 indicates where 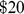 incentives were used and 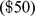 for 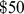 incentives. The vaccination programs are the passive (PV), along with the pro-active programs: random vaccination (RV), nearest neighbor (NN), chain (CV) and improved nearest neighbor (INN).(PDF)

Table S3The global clustering coefficient among susceptible individuals (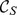) and the pair correlations between susceptible and infected individuals (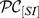) and susceptible and vaccinated individuals (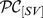) for the various vaccination strategies.(PDF)

Table S4The statistics regarding the number of superspreaders that were willing to vaccinate prior to being recruited (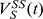), that were randomly contacted and then were willing to vaccinate (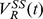) and those contacted through a nearest neighbor and then were willing to vaccinate (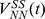) for the various vaccination strategies (with and without incentives) (empirically-based network). 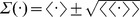, where 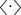 denotes the average and 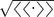 denotes the standard deviation. NB indicates the scenario where vaccination behavior is entirely ignored, 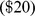 indicates where 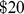 incentives were used and 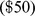 for 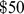 incentives. The vaccination programs are the passive (PV), along with the pro-active programs: random vaccination (RV), nearest neighbor (NN), chain (CV) and improved nearest neighbor (INN).(PDF)

Table S5The estimated costs of the various vaccination strategies for the Realistic networks. The vaccination programs are the passive, along with the pro-active programs: random vaccination (RV), nearest neighbor (NN), chain (CV) and improved nearest neighbor (INN). 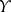 indicates an incentive value of 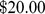, where 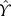 indicates an incentive value of 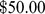. 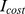 refers to the cost of infection, 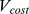 refers to the cost of vaccination and 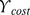 refers to the cost associated with incentives. 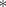 the strategy was slightly altered to only allow the incentives to be distributed to the acquaintance.(PDF)

Table S6Influenza incidence and vaccine coverage for the various vaccination strategies (with and without incentives) where there is heterogeneity in the infectious period and transmission rate (exponential network). 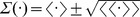, where 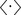 denotes the average and 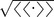 denotes the standard deviation. The annual incidence is denoted by 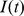, where 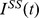 denotes the annual incidence of the superspreading population. The annual vaccine uptake is denoted as 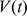, where the vaccine uptake in the superspreading population is denoted as 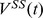. NB indicates the scenario where vaccination behavior is entirely ignored, 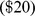 indicates where 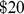 incentives were used and 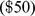 for 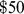 incentives. The vaccination programs are the passive (PV), along with the pro-active programs: random vaccination (RV), nearest neighbor (NN), chain (CV) and improved nearest neighbor (INN).(PDF)

Table S7Influenza incidence and vaccine coverage for the various vaccination strategies (with and without incentives) where there is heterogeneity in the infectious period and transmission rate (Poisson network). 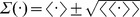, where 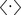 denotes the average and 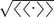 denotes the standard deviation. The annual incidence is denoted by 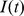, where 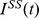 denotes the annual incidence of the superspreading population. The annual vaccine uptake is denoted as 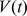, where the vaccine uptake in the superspreading population is denoted as 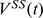. NB indicates the scenario where vaccination behavior is entirely ignored, 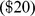 indicates where 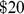 incentives were used and 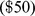 for 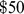 incentives. The vaccination programs are the passive (PV), along with the pro-active programs: random vaccination (RV), nearest neighbor (NN), chain (CV) and improved nearest neighbor (INN).(PDF)

Text S1 The supplementary text provides further detailed information pertaining to the modelling of disease dynamics and vaccination behavior, the classification of a superspreader and the various vaccination strategies.
(PDF)
