# Supplementary figures and images for: Policy Resistance Undermines Superspreader Vaccination Strategies for Influenza
Source: PLoS Comput Biol. 2013 Mar 7;9(3):e1002945. doi: 10.1371/journal.pcbi.1002945 (PMC3591296; doi:10.1371/journal.pcbi.1002945)

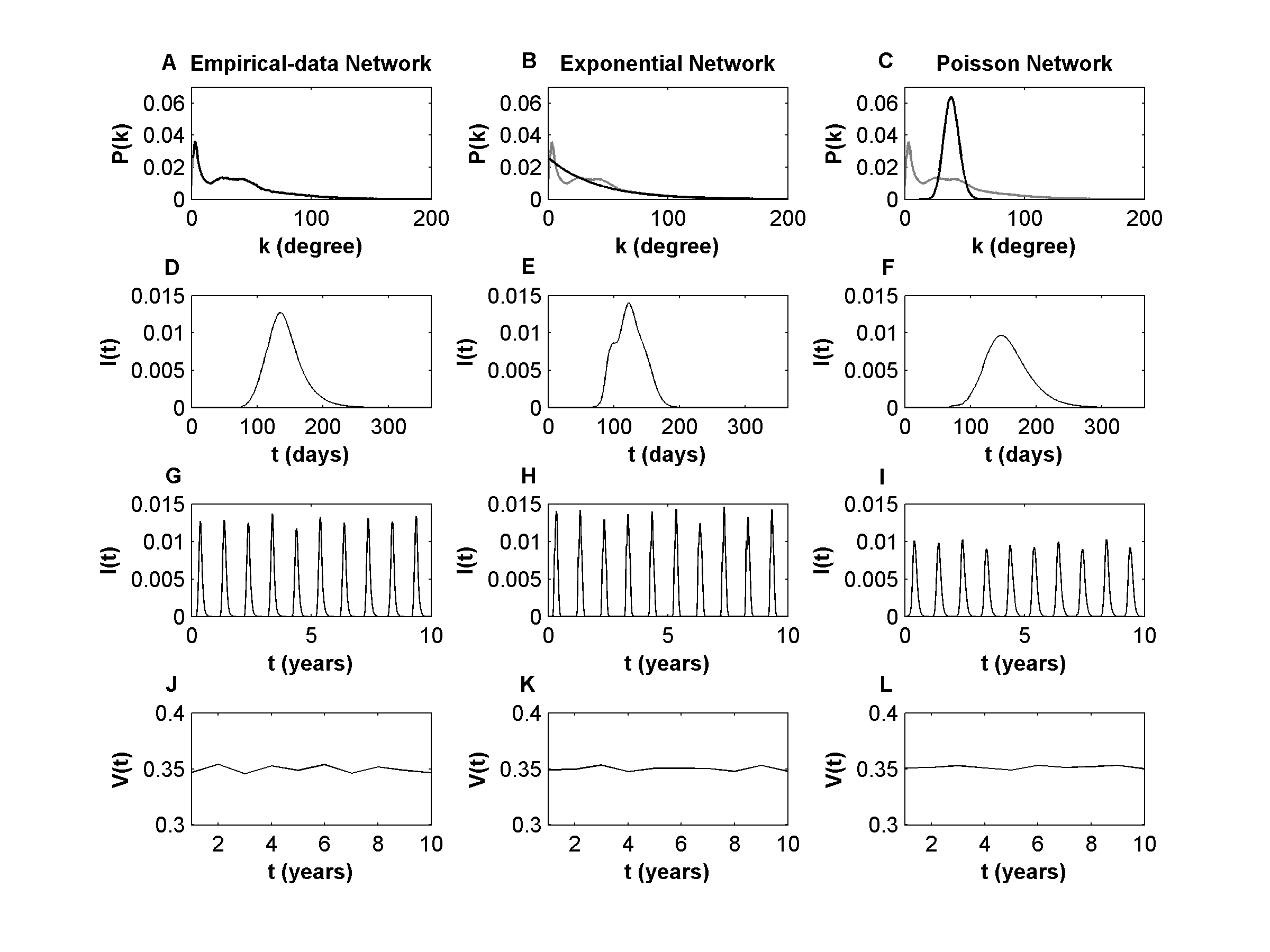

Supplement: Figure S1 — The top row, a)–c), shows the degree distribution for each network (black) compared to the original empirical network (gray) of Portland, Oregon [32]–[34]. The second row, d)–f), clearly shows that prevalence peaks between the beginning of January and the end of February. The third row, g)–i), shows the prevalence over many years under no vaccination and the fourth row, j)–l), shows the average vaccine coverage over the years under passive vaccination. The average time for peak of prevalence: Empirically-based , Exponential and Poisson . The approximate average duration of the season: Empirically-based days, Exponential days and Poisson days. (TIFF) [file pcbi.1002945.s001.tiff]
