# Supplementary material for: Policy Resistance Undermines Superspreader Vaccination Strategies for Influenza
Source: PLoS Comput Biol. 2013 Mar 7;9(3):e1002945. doi: 10.1371/journal.pcbi.1002945 (PMC3591296; doi:10.1371/journal.pcbi.1002945)
Supplement: Table S1 — The values and descriptions of the parameters used in the simulations for the Poisson and exponential networks. The values were calibrated for each network using the passive vaccination approach. The values , and were calibrated such that the average annual vaccine coverage on each network was approximately using appropriate values; the monthly vaccine uptake was based on the 2010–2011 influenza season [67]. was used in calibrating influenza incidence () using values similar to influenza's [40]–[42] on each network such that the average peak of prevalence occurred between January and February [66]. (PDF) [file pcbi.1002945.s003.pdf]

Parameter	Description	Value	Reference
$\langle k \rangle$	Average node degree for networks	39	[1-3]
$\mathcal{R}_0^P$	Null Deterministic Basic Reproductive Value (Poisson)	2.2	calibrated $**$;[4-7]
$\mathcal{R}_0^E$	Null Deterministic Basic Reproductive Value (exponential)	2.875	calibrated $**$;[4-7]
p_s	Change in Seasonality Amplitude (Poisson)	0.03	[5,8]
p_s	Change in Seasonality Amplitude (exponential)	0.0525	[5,8]
$\bar{t}$	Shift in Seasonality function (Poisson)	76	calibrated$**$
$\bar{t}$	Shift in Seasonality function (exponential)	122	calibrated $**$
$\bar{I}$	Number of Exogenous Infections (Poisson)	7	calibrated $**$
$\bar{I}$	Number of Exogenous Infections (exponential)	7	calibrated $**$
b	Parameter for vaccine uptake equation (Poisson)	1360	calibrated
b	Parameter for vaccine uptake equation (exponential)	4000	calibrated
$\langle \phi \rangle_{SEPT}$	Average vaccine uptake for September	0.0593	[9]
$\langle\langle \phi \rangle\rangle_{SEPT}$	Variance in vaccine uptake for September	0.00015	[9]
$\langle \phi \rangle_{OCT}$	Average vaccine uptake for October	0.1842	[9]
$\langle\langle \phi \rangle\rangle_{OCT}$	Variance in vaccine uptake for October	0.00072	[9]
$\langle \phi \rangle_{NOV}$	Average vaccine uptake for November	0.1012	[9]
$\langle\langle \phi \rangle\rangle_{NOV}$	Variance in vaccine uptake for November	0.00038	[9]
$\langle \phi \rangle_{DEC}$	Average vaccine uptake for December	0.0238	[9]
$\langle\langle \phi \rangle\rangle_{DEC}$	Variance in vaccine uptake for December	0.00004	[9]
