# Supplementary material for: Policy Resistance Undermines Superspreader Vaccination Strategies for Influenza
Source: PLoS Comput Biol. 2013 Mar 7;9(3):e1002945. doi: 10.1371/journal.pcbi.1002945 (PMC3591296; doi:10.1371/journal.pcbi.1002945)
Supplement: Table S2 — Percentage of recruitments where recruitment did not matter, whether due to infection, already vaccinated or will be vaccinating in the future (Useless) and the percentage of incentives actually used ( Used). NB indicates the scenario where vaccination behavior is entirely ignored, indicates where incentives were used and for incentives. The vaccination programs are the passive (PV), along with the pro-active programs: random vaccination (RV), nearest neighbor (NN), chain (CV) and improved nearest neighbor (INN). (PDF) [file pcbi.1002945.s004.pdf]

Strategy	% Useless	%Υ Used
PV + RV	36.82%	*N/A*
PV + NN	37.43%	*N/A*
PV + CV	37.63%	*N/A*
PV + INN	38.58%	*N/A*
PV + RV (NB)	43.49%	*N/A*
PV + NN (NB)	44.63%	*N/A*
PV + CV (NB)	45.08%	*N/A*
PV + INN (NB)	47.38%	*N/A*
PV + RV ($20)	37.11%	57.02%
PV + NN ($20)	37.57%	56.61%
PV + NN ($20)$*$	37.34%	57.12%
PV + CV ($20)	38.01%	57.01%
PV + INN ($20)	39.31%	56.29%
PV + INN ($20)$*$	39.18%	57.10%
PV + RV ($50)	37.39%	71.11%
PV + NN ($50)	37.73%	70.77%
PV + NN ($50)$*$	37.33%	70.62%
PV + CV ($50)	38.34%	70.86%
PV + INN ($50)	39.85%	69.58%
PV + INN ($50)$t$	39.49%	67.89%
