# Supplementary material for: Policy Resistance Undermines Superspreader Vaccination Strategies for Influenza
Source: PLoS Comput Biol. 2013 Mar 7;9(3):e1002945. doi: 10.1371/journal.pcbi.1002945 (PMC3591296; doi:10.1371/journal.pcbi.1002945)
Supplement: Table S3 — The global clustering coefficient among susceptible individuals () and the pair correlations between susceptible and infected individuals () and susceptible and vaccinated individuals () for the various vaccination strategies. (PDF) [file pcbi.1002945.s005.pdf]

Strategy	$\mathcal{C}_S$	$\mathcal{PC}_{[SI]}$	$\mathcal{PC}_{[SV]}$
PV	0.606	0.78	0.69
PV + RV	0.607	0.79	0.69
PV + NN	0.609	0.78	0.70
PV + CV	0.608	0.77	0.71
PV + INN	0.613	0.77	0.72
PV (NB)	0.600	0.82	0.68
PV + RV (NB)	0.594	0.88	0.74
PV + NN (NB)	0.603	0.84	0.77
PV + CV (NB)	0.603	0.83	0.77
PV + INN (NB)	0.612	0.80	0.79
PV + RV ($20)	0.604	0.81	0.70
PV + NN ($20)	0.611	0.78	0.73
PV + CV ($20)	0.610	0.77	0.73
PV + INN ($20)	0.619	0.75	0.75
PV + RV ($50)	0.602	0.83	0.71
PV + NN ($50)	0.611	0.78	0.74
PV + CV ($50)	0.610	0.78	0.74
PV + INN ($50)	0.620	0.75	0.77
