# Supplementary material for: Policy Resistance Undermines Superspreader Vaccination Strategies for Influenza
Source: PLoS Comput Biol. 2013 Mar 7;9(3):e1002945. doi: 10.1371/journal.pcbi.1002945 (PMC3591296; doi:10.1371/journal.pcbi.1002945)
Supplement: Table S4 — The statistics regarding the number of superspreaders that were willing to vaccinate prior to being recruited (), that were randomly contacted and then were willing to vaccinate () and those contacted through a nearest neighbor and then were willing to vaccinate () for the various vaccination strategies (with and without incentives) (empirically-based network). , where denotes the average and denotes the standard deviation. NB indicates the scenario where vaccination behavior is entirely ignored, indicates where incentives were used and for incentives. The vaccination programs are the passive (PV), along with the pro-active programs: random vaccination (RV), nearest neighbor (NN), chain (CV) and improved nearest neighbor (INN). (PDF) [file pcbi.1002945.s006.pdf]

Strategy	$\Sigma(V_S^{SS}(t))$	$\Sigma(V_R^{SS}(t))$	$\Sigma(V_{NN}^{SS}(t))$
PV	0.39 ± 0.062	0 ± 0	0 ± 0
PV + RV	0.37 ± 0.059	0.041 ± 0.0063	0 ± 0
PV + NN	0.37 ± 0.059	0.02 ± 0.0044	0.051 ± 0.007
PV + CV	0.37 ± 0.059	0.02 ± 0.0043	0.051 ± 0.0071
PV + INN	0.36 ± 0.058	0.019 ± 0.0042	0.081 ± 0.0087
PV (NB)	0.35 ± 0.015	0 ± 0	0 ± 0
PV + RV (NB)	0.35 ± 0.014	0.14 ± 0.01	0 ± 0
PV + NN (NB)	0.35 ± 0.015	0.063 ± 0.0071	0.16 ± 0.011
PV + CV (NB)	0.35 ± 0.015	0.063 ± 0.0073	0.16 ± 0.011
PV + INN (NB)	0.36 ± 0.014	0.058 ± 0.0068	0.22 ± 0.012
PV + RV ($20)	0.35 ± 0.054	0.094 ± 0.0088	0 ± 0
PV + NN ($20)	0.33 ± 0.052	0.045 ± 0.0061	0.11 ± 0.0094
PV + CV ($20)	0.33 ± 0.051	0.045 ± 0.0063	0.11 ± 0.01
PV + INN ($20)	0.32 ± 0.049	0.043 ± 0.0061	0.18 ± 0.011
PV + RV ($50)	0.33 ± 0.048	0.13 ± 0.012	0 ± 0
PV + NN ($50)	0.31 ± 0.044	0.061 ± 0.0077	0.15 ± 0.013
PV + CV ($50)	0.31 ± 0.045	0.061 ± 0.0079	0.15 ± 0.014
PV + INN ($50)	0.29 ± 0.042	0.058 ± 0.0075	0.23 ± 0.016
