# Supplementary material for: Policy Resistance Undermines Superspreader Vaccination Strategies for Influenza
Source: PLoS Comput Biol. 2013 Mar 7;9(3):e1002945. doi: 10.1371/journal.pcbi.1002945 (PMC3591296; doi:10.1371/journal.pcbi.1002945)
Supplement: Table S5 — The estimated costs of the various vaccination strategies for the Realistic networks. The vaccination programs are the passive, along with the pro-active programs: random vaccination (RV), nearest neighbor (NN), chain (CV) and improved nearest neighbor (INN). indicates an incentive value of , where indicates an incentive value of . refers to the cost of infection, refers to the cost of vaccination and refers to the cost associated with incentives. the strategy was slightly altered to only allow the incentives to be distributed to the acquaintance. (PDF) [file pcbi.1002945.s007.pdf]

Strategy	I_{cost}	V_{cost}	Υ_{cost}	Total
PV	$101,123.45	$69,968.98	$0.00	$171,092.43
PV + RV	$96,814.46	$75,163.34	$0.00	$171,977.80
PV + NN	$95,974.89	$74,307.91	$0.00	$170,282.80
PV + CV	$95,994.61	$74,293.25	$0.00	$170,287.86
PV + INN	$95,351.87	$73,443.82	$0.00	$168,795.69
PV (NB)	$97,708.81	$70,239.57	$0.00	$167,948.38
PV + RV (NB)	$75,403.22	$97,665.35	$0.00	$173,068.57
PV + NN (NB)	$70,053.29	$97,116.51	$0.00	$167,169.80
PV + CV (NB)	$70,399.56	$96,870.48	$0.00	$167,270.04
PV + INN (NB)	$66,465.58	$95,767.50	$0.00	$162,233.08
PV + RV $20	$91,776.33	$81,049.27	$27,827.02	$200,652.62
PV + NN $20	$89,939.64	$79,087.34	$27,626.24	$196,653.22
PV + NN $20∗	$92,470.28	$75,945.11	$13,937.53	$182,352.91
PV + CV $20	$90,135.70	$79,035.00	$27,822.40	$196,993.10
PV + INN $20	$88,335.36	$76,943.90	$27,467.32	$192,746.58
PV + INN $20∗	$90,924.69	$73,885.44	$13,932.77	$178,742.90
PV + RV $50	$87,977.25	$85,177.45	$86,753.29	$259,907.99
PV + NN $50	$85,070.74	$82,608.28	$86,342.32	$254,021.34
PV + NN $50∗	$89,966.28	$77,068.94	$43,078.52	$210,113.74
PV + CV $50	$85,482.37	$82,491.13	$86,451.52	$254,425.02
PV + INN $50	$83,209.41	$79,930.13	$84,885.34	$248,024.88
PV + INN $50∗	$88,015.02	$74,140.18	$41,410.88	$203,566.08
