# Supplementary material for: Policy Resistance Undermines Superspreader Vaccination Strategies for Influenza
Source: PLoS Comput Biol. 2013 Mar 7;9(3):e1002945. doi: 10.1371/journal.pcbi.1002945 (PMC3591296; doi:10.1371/journal.pcbi.1002945)
Supplement: Table S6 — Influenza incidence and vaccine coverage for the various vaccination strategies (with and without incentives) where there is heterogeneity in the infectious period and transmission rate (exponential network). , where denotes the average and denotes the standard deviation. The annual incidence is denoted by , where denotes the annual incidence of the superspreading population. The annual vaccine uptake is denoted as , where the vaccine uptake in the superspreading population is denoted as . NB indicates the scenario where vaccination behavior is entirely ignored, indicates where incentives were used and for incentives. The vaccination programs are the passive (PV), along with the pro-active programs: random vaccination (RV), nearest neighbor (NN), chain (CV) and improved nearest neighbor (INN). (PDF) [file pcbi.1002945.s008.pdf]

Strategy	$\Sigma(I(t))$	$\Sigma(V(t))$	$\Sigma(I^{SS}(t))$	$\Sigma(V^{SS}(t))$
No Vaccination	0.15 ± 0.087	0 ± 0	0.22 ± 0.13	0 ± 0
PV	0.1 ± 0.077	0.35 ± 0.059	0.16 ± 0.12	0.38 ± 0.07
PV + RV	0.096 ± 0.074	0.37 ± 0.059	0.16 ± 0.12	0.4 ± 0.07
PV + NN	0.095 ± 0.075	0.37 ± 0.059	0.16 ± 0.12	0.42 ± 0.072
PV + CV	0.095 ± 0.075	0.37 ± 0.059	0.16 ± 0.12	0.42 ± 0.071
PV + INN	0.095 ± 0.075	0.36 ± 0.058	0.15 ± 0.12	0.44 ± 0.072
PV (NB)	0.097 ± 0.06	0.35 ± 0.0049	0.16 ± 0.1	0.35 ± 0.014
PV + RV (NB)	0.077 ± 0.051	0.49 ± 0.0053	0.14 ± 0.092	0.49 ± 0.015
PV + NN (NB)	0.071 ± 0.05	0.48 ± 0.0055	0.13 ± 0.09	0.56 ± 0.016
PV + CV (NB)	0.071 ± 0.051	0.48 ± 0.0055	0.13 ± 0.09	0.56 ± 0.016
PV + INN (NB)	0.069 ± 0.05	0.47 ± 0.0054	0.12 ± 0.086	0.59 ± 0.016
PV + RV ($20)	0.092 ± 0.071	0.41 ± 0.054	0.15 ± 0.12	0.43 ± 0.064
PV + NN ($20)	0.089 ± 0.071	0.39 ± 0.052	0.15 ± 0.11	0.47 ± 0.062
PV + CV ($20)	0.089 ± 0.07	0.39 ± 0.051	0.15 ± 0.11	0.47 ± 0.061
PV + INN ($20)	0.088 ± 0.07	0.38 ± 0.05	0.14 ± 0.11	0.5 ± 0.059
PV + RV ($50)	0.088 ± 0.065	0.43 ± 0.038	0.15 ± 0.11	0.45 ± 0.049
PV + NN ($50)	0.084 ± 0.064	0.41 ± 0.037	0.14 ± 0.11	0.51 ± 0.043
PV + CV ($50)	0.085 ± 0.065	0.41 ± 0.037	0.14 ± 0.11	0.5 ± 0.044
PV + INN ($50)	0.083 ± 0.064	0.4 ± 0.036	0.14 ± 0.1	0.53 ± 0.041
