# Supplementary material for: Policy Resistance Undermines Superspreader Vaccination Strategies for Influenza
Source: PLoS Comput Biol. 2013 Mar 7;9(3):e1002945. doi: 10.1371/journal.pcbi.1002945 (PMC3591296; doi:10.1371/journal.pcbi.1002945)
Supplement: Table S7 — Influenza incidence and vaccine coverage for the various vaccination strategies (with and without incentives) where there is heterogeneity in the infectious period and transmission rate (Poisson network). , where denotes the average and denotes the standard deviation. The annual incidence is denoted by , where denotes the annual incidence of the superspreading population. The annual vaccine uptake is denoted as , where the vaccine uptake in the superspreading population is denoted as . NB indicates the scenario where vaccination behavior is entirely ignored, indicates where incentives were used and for incentives. The vaccination programs are the passive (PV), along with the pro-active programs: random vaccination (RV), nearest neighbor (NN), chain (CV) and improved nearest neighbor (INN). (PDF) [file pcbi.1002945.s009.pdf]

Strategy	$\Sigma(I(t))$	$\Sigma(V(t))$	$\Sigma(I^{SS}(t))$	$\Sigma(V^{SS}(t))$
No Vaccination	0.15 ± 0.12	0 ± 0	0.15 ± 0.13	0 ± 0
PV	0.065 ± 0.07	0.35 ± 0.03	0.069 ± 0.078	0.35 ± 0.04
PV + RV	0.056 ± 0.062	0.38 ± 0.028	0.06 ± 0.069	0.38 ± 0.038
PV + NN	0.056 ± 0.062	0.38 ± 0.028	0.06 ± 0.069	0.38 ± 0.038
PV + CV	0.056 ± 0.062	0.38 ± 0.028	0.06 ± 0.069	0.38 ± 0.038
PV + INN	0.056 ± 0.061	0.38 ± 0.028	0.059 ± 0.068	0.38 ± 0.038
PV (NB)	0.059 ± 0.059	0.35 ± 0.005	0.063 ± 0.066	0.35 ± 0.025
PV + RV (NB)	0.022 ± 0.025	0.49 ± 0.0046	0.024 ± 0.03	0.49 ± 0.026
PV + NN (NB)	0.022 ± 0.024	0.49 ± 0.0045	0.023 ± 0.029	0.49 ± 0.026
PV + CV (NB)	0.022 ± 0.025	0.49 ± 0.0046	0.023 ± 0.029	0.49 ± 0.026
PV + INN (NB)	0.021 ± 0.024	0.49 ± 0.0046	0.022 ± 0.028	0.5 ± 0.026
PV + RV ($20)	0.052 ± 0.058	0.39 ± 0.026	0.056 ± 0.065	0.39 ± 0.036
PV + NN ($20)	0.054 ± 0.06	0.38 ± 0.027	0.058 ± 0.067	0.39 ± 0.037
PV + CV ($20)	0.052 ± 0.058	0.39 ± 0.026	0.056 ± 0.065	0.39 ± 0.037
PV + INN ($20)	0.051 ± 0.058	0.39 ± 0.026	0.055 ± 0.064	0.4 ± 0.036
PV + RV ($50)	0.046 ± 0.051	0.41 ± 0.021	0.049 ± 0.057	0.41 ± 0.033
PV + NN ($50)	0.05 ± 0.056	0.39 ± 0.025	0.054 ± 0.063	0.4 ± 0.035
PV + CV ($50)	0.046 ± 0.051	0.41 ± 0.021	0.049 ± 0.057	0.42 ± 0.033
PV + INN ($50)	0.045 ± 0.05	0.41 ± 0.021	0.048 ± 0.056	0.42 ± 0.033
