# Supplementary material for: Policy Resistance Undermines Superspreader Vaccination Strategies for Influenza
Source: PLoS Comput Biol. 2013 Mar 7;9(3):e1002945. doi: 10.1371/journal.pcbi.1002945 (PMC3591296; doi:10.1371/journal.pcbi.1002945)
Supplement: Text S1 — The supplementary text provides further detailed information pertaining to the modelling of disease dynamics and vaccination behavior, the classification of a superspreader and the various vaccination strategies. (PDF) [file pcbi.1002945.s010.pdf]

Supplementary Text: Policy resistance undermines superspreader vaccination strategies for influenza

Chad R. Wells [1,*], Eili Y. Klein2[2], Chris T. Bauch[1]
1 Department of Mathematics and Statistics, University of Guelph, Guelph, Ontario, Canada
2 Center for Advanced Modeling, Department of Emergency Medicine, Johns Hopkins University, Baltimore, Maryland, USA
∗ E-mail: cwells01@uoguelph.ca

Disease Dynamics

Additional baseline parameter values appear in Table S1, along with literature sources [1–12]. We assumed a Susceptible-Infected-Recovered-Vaccinated-Susceptible ($SIRVS$) natural history and voluntary vaccination. A susceptible individual becomes infected ($S \to I$) by a single infectious contact with probability $p(t)$ per day, which varies seasonally. The seasonality probability of transmission is represented as

$$p(t) = \langle p \rangle \left(1 + p_s \sin\left(\frac{2\pi(t-\bar{t})}{365}\right)\right) \qquad (1)$$

where $p(t)$ is the probability (per day) of infection passing along the edge connecting an infected individual and a susceptible individual, $\langle p \rangle$ is the average transmission rate from an infected individual to a single susceptible individual per day and the constant $p_s \in (0,1)$ represents the impact of seasonality on the rate of transmission (i.e the change in the seasonal transmission amplitude). An infectious individual moves to the recovered state ($I \to R$) after δ days. A recovered individual becomes susceptible ($R \to S$) with probability ρ per year (natural immunity). A vaccinated individual becomes susceptible ($V \to S$) with probability ω per year (vaccine immunity). We assume that vaccination has no impact on individuals who are infectious or recovered. We assume an individual does not attain vaccine immunity if they have already obtained natural immunity.

We assume non-influenzal influenza-like-illness (niILI) is also present, where niILI is randomly distributed throughout the network daily for the entire year. The probability of an individual experiencing niILI daily is denoted by α, where α is sampled from a log-normal distribution with mean $\langle \alpha \rangle$ and variance $\langle\langle \alpha \rangle\rangle$,

$$\alpha \sim \ln \mathcal{N}(\mu, \sigma^2) \qquad (2)$$

$$\mu = \ln(\langle \alpha \rangle) - \frac{1}{2}\ln\left(1 + \frac{\langle\langle \alpha \rangle\rangle}{\langle \alpha \rangle^2}\right) \qquad (3)$$

$$\sigma^2 = \ln\left(1 + \frac{\langle\langle \alpha \rangle\rangle}{\langle \alpha \rangle^2}\right), \qquad (4)$$

where μ and σ^2 represent the mean and variance of the Normal distribution (i.e. $\ln(\alpha) \sim \mu + \sigma\mathcal{Z}$, where $\mathcal{Z} \sim \mathcal{N}(0,1)$). We assume an individual mistakes niILI for true influenza with probability β.

We assume infection is introduced slowly into the population starting in mid-November, where a randomly chosen individual will be infected every few weeks. The main reason for the exogenous introduction of infection each year is to compensate for stochastic extinction that may occur in the network in the summer months. Infection is introduced in mid-November such that the peak of infection corresponds to a time between the start of January and end of February [12]. A typical influenza season generally starts approximately at the end of October/mid part of November and lasts until the end of April/middle of May [13]. The infection parameter values for equation (1) were calibrated to obtain incidence of approximately 15% [4,10,11] for each network (Poisson, exponential and empirically-based), under the condition of no vaccination (Table 1 and Table S1).

The average transmission rate among individuals is $\langle p\rangle = q/\langle k\rangle$, where q is the average number of people infected per day and $\langle k\rangle$ is the average degree of the network. For the empirically-based network, the value of $\langle k\rangle$ was taken to be the minimum average node degree of the 10 empirically-based networks, such that, $\langle p\rangle$ would be sufficiently large enough to produce an epidemic in all of the empirically-based networks. The simulation started at $t = 0$. The days that the exogenous infections were introduced for the Poisson and exponential networks were: $t = 68, 89, 96, 99, 117, 119$ and 121; for the empirically-based network this was: $t = 75, 89, 97, 103, 110, 117, 118, 119, 120, 121$ and 122. We increased the frequency of infection near the end of the sequence to increase the force of infection such that the average peak would occur at a realistic time of year. In the exponential network, the first four exogenous infections only infected nodes of degree one or two; this allowed the infection to spread at a slower rate and not peak as quickly. See Figure S1 for the results of the calibration regarding prevalence in each of the networks.

Classifying Super-Spreaders

A super-spreader is classified as an individual who infects more people than the 95th percentile from a Poisson distribution with mean $\mathcal{R}_0$; $\mathcal{R}_0$ is the basic reproduction value from the "null" deterministic model [14, 15]. The "null" deterministic model is

$$\frac{dS}{dt} = \bar{\rho}R - \frac{qSI}{N} \qquad (5)$$

$$\frac{dI}{dt} = \frac{qSI}{N} - \bar{\delta}I \qquad (6)$$

$$\frac{dR}{dt} = \bar{\delta}I - \bar{\rho}R \qquad (7)$$

where $\bar{\delta} = 1/\delta$ (δ is the average infectious period) is the rate (per day) at which a single infectious individual moves to the recovered state ($I \to R$) and $\bar{\rho} = 1/(365\rho)$ (ρ is the natural waning immunity in years) is the rate (per day) at which a recovered individual becomes susceptible ($R \to S$). The basic reproductive value for the "null" deterministic model is

$$\mathcal{R}_0 = \frac{q}{\bar{\delta}} = q\delta. \qquad (8)$$

The value q/N represents the probability that a single infectious individual infects a susceptible individual, i.e. $\langle p\rangle = q/N$. However, in our network model an individual only has a given number of contacts and the transmission rate can vary between individuals. We assume the number of daily infections, q, can be approximated by the average transmission rate $\langle p\rangle$ and the average degree $\langle k\rangle$, where

$$q = \langle p\rangle\langle k\rangle. \qquad (9)$$

Our assumption comes from the fact that a network's $\mathcal{R}_0$ approaches the null deterministic model's $\mathcal{R}_0$ as $\langle k\rangle$ increases, where$\langle p\rangle\langle k\rangle \to q$, while keeping q constant [16].

We assume the basic reproduction value for an individual is

$$\begin{aligned}\mathcal{R}_0 &= \sum_{i=1}^{k} \binom{k}{i} i(1-(1-\langle p\rangle)^{\delta})^{i}(1-\langle p\rangle)^{\delta(k-i)} \qquad (10)\\ &= k(1-(1-\langle p\rangle)^{\delta}),\end{aligned}$$

where $(1-\langle p\rangle)^{\delta}$ is the probability that an individual is not infected for δ days; therefore, $(1-(1-\langle p\rangle)^{\delta})$ is the probability that an individual is infected during the δ days, k is the number of contacts of the infected individual and δ is the infectious period.

There are three possible sources of super-spreading that we focus on:

1. a large number of contacts
2. increased infectious period
3. increased transmission of the disease

We consider four separate cases of super-spreading 1) heterogeneity in node degree 2) heterogeneity in node degree and infectious period 3) heterogeneity in node degree and transmission rate and 4) heterogeneity in node degree, infectious period and transmission rate.

Super-spreader: Number of Contacts

In the case of the super spreader having a large number of contacts, social contact networks will be used. For simple network analysis our first choice is a network with a Poisson degree distribution, where the cumulative distribution function is

$$d(k) = \sum_{n=0}^{k} \frac{\langle k \rangle^n e^{-\langle k \rangle}}{n!} \tag{11}$$

Social contact networks have been shown to be well represented by an exponential degree distribution [17], where the cumulative distribution function for the exponential distribution is

$$d(k) = 1 - e^{-k/\langle k \rangle} \ (k > 0) \tag{12}$$

For influenza, the exponential network falls in between the unrealistic power-law and unrealistic Poisson. The average degree ($\langle k \rangle$) for both the Poisson and exponential networks was obtained from the empirical data from Ref [1–3], describing Portland, Oregon.

We also created scaled down empirically-based networks using data from Portland, Oregon [1–3]. To scale down the Portland network, we randomly selected an individual, recorded their contacts, then recorded their contact's contacts and so on, until the desired population size was reached. We then rewired the network such that the degree distribution of the sampled network resembled the original empirical degree distribution. We produced 10 Portland-like networks for our sample.

We assumed an individual's infectious period (δ), transmission ($\langle p \rangle$), natural waning immunity (ρ), vaccine waning immunity (ω) come from a delta-distribution (i.e. there is no heterogeneity). The reason for this approach is it allows for the impact of the different networks to be analyzed without having to understand the impact of extra parameter sampled from distributions. The average transmission will be calculated by

$$\langle p \rangle = \frac{q}{\langle k \rangle}, \tag{13}$$

where $\langle k \rangle$ is the average degree in the network. The degree distributions for each network can be seen in Figure S1.

Super-spreader: Increased Infectious Period & Number of Contacts

In the case of an increased infectious period, we assumed an individual's infectious period is described by a Poisson distribution with mean δ, and remains that value throughout the simulation. We resampled any infectious period that was zero or too large; i.e. for the case where $\delta = 5$, we resampled any infectious period larger than 14 days. We ran this scenario on the three networks (Poisson, exponential and empirically-based) and assumed all other parameters come from a delta distribution.

Super-spreader: Increased Transmission & Number of Contacts

In the case of an increased transmission probability, we assume an individual's average transmission is provided by a log-normal distribution with mean $\langle p \rangle$, which remains static through a simulation. We assumed a skewed log-normal distribution to allow for extreme cases of super-spreading in some individuals. We ran this scenario on the three networks (Poisson, exponential and empirically-based) and assumed all other parameters come from a delta distribution.

Super-spreader: Heterogeneous Population

We assumed heterogeneity in the number of contacts, infection period and transmission. We assumed the infectious period is sampled from a Poisson distribution with mean δ, the transmission rate is sampled from a log-normal distribution with mean $\langle p \rangle$ and the network is created from the three networks discussed earlier, where all other parameters come from a delta distribution.

Probability of Vaccination

An individual first determines whether or not they will seek vaccination for this upcoming season or not, based upon their payoffs. The probability that an individual seeks vaccination for this upcoming season is

$$\Phi(\mathcal{P}_{\mathcal{V}} - \mathcal{P}_{\mathcal{N}}) = \frac{\arctan\left(b(\mathcal{P}_{\mathcal{V}} - \mathcal{P}_{\mathcal{N}})/(\$/QALY)\right) + \frac{\pi}{2}}{\pi}, \tag{14}$$

The value of b in equation (14) was used to calibrate the vaccine coverage of the model, along with the dollar per Quality Adjusted Life Year (QALY) ($\$/QALY$). Each value of b for the corresponding network is given in Table 1 and Table S1. As the difference in payoffs, $\mathcal{P}_{\mathcal{V}} - \mathcal{P}_{\mathcal{N}}$, increases (decreases) the more (less) likely the individual will seek vaccination this season, since vaccination becomes more (less) appealing. If incentives are included, we use $\Phi(\mathcal{P}_{\mathcal{V}} - \mathcal{P}_{\mathcal{N}} + \Upsilon n)$ in determining whether or not the individual seeks vaccination this season, where Υ is the value of the monetary incentive and n is the number of incentives received.

Once it is determined that an individual is going to vaccinate, the following process is used to determine when the individual vaccinates. Prior to the new vaccination season we determine the vaccine uptake for each month, ϕ_{month}, by sampling from a log-normal distribution with a mean of $\langle \phi \rangle$ and a variance of $\langle\langle \phi \rangle\rangle$ (Table 1 and Table S1), that is

$$\begin{aligned} \phi_{month} &\sim \ln\mathcal{N}(\mu, \sigma^2) & (15)\\ \mu &= \ln(\langle \phi_{month} \rangle) - \frac{1}{2}\ln\left(1 + \frac{\langle\langle \phi_{month} \rangle\rangle}{\langle \phi_{month} \rangle^2}\right) & (16)\\ \sigma^2 &= \ln\left(1 + \frac{\langle\langle \phi_{month} \rangle\rangle}{\langle \phi_{month} \rangle^2}\right). & (17) \end{aligned}$$

With these values we can determine when the individual vaccinates. We start off by selecting a uniform random number $0 < r < 1$, then find the first month such that

$$r\left(\sum_{\forall j} \phi_j\right) < \sum_{j \le month} \phi_j, \tag{18}$$

Once a month is determined for the individual, a day is then chosen at random from that month for the individual to vaccinate on.

If the individual is seeking vaccination, they will only vaccinate if they have not experienced influenza in the current season; experiencing influenza includes cases of niILI being mistaken for influenza and excludes non-symptomatic cases of influenza. The average annual vaccine coverage can be seen in Figure S1.

Social Influence

We incorporate social influence by using an imitation process or learning process; the learning process consists of individuals exchanging information regarding time since last infection, time since last complication and perceived vaccine efficacy. The learning process occurs with a probability σ, which we call the imitation strength; the process consists of individual i randomly selecting one of their contacts, individual j, as well as randomly selecting an individual from the network, individual k. Individual i then updates their informations as follows

$$\begin{aligned} {T_I}^{i_{new}} &= (1-\sigma){T_I}^i + \sigma(\nu {T_I}^j + (1-\nu){T_I}^k) && (19) \\ {T_C}^{i_{new}} &= (1-\sigma){T_C}^i + \sigma(\nu {T_C}^j + (1-\nu){T_C}^k) && (20) \\ \varepsilon^{i_{new}}(t) &= (1-\sigma)\varepsilon^i(t) + \sigma(\nu\varepsilon^j(t) + (1-\nu)\varepsilon^k(t)), && (21) \end{aligned}$$

where the super-script denotes the individual and ν denotes the preference of using a contacts information to the randomly selected individual (i.e. $\nu = 1$ the individual prefers to imitate their contact). This corresponds to the sampler internalizing the experience of the sampled individual.

Pro-Active Vaccination Policies

We focus on four pro-active approaches public health could implement in society; we are assuming public health will contact $\bar{\mathcal{V}}$ individuals per day and recommend vaccination.

In each pro-active policy, if the selected individual decided not to vaccinate at the beginning of the season they proceed through the learning (imitation) process again and determine whether or not they will seek vaccination, using equation (14), with their new updated payoffs. The individual will decide when to vaccinate using an approach similar to that in equation (18); however, if the month has passed, it is no longer used in determining when the individual will vaccinate. That is, if September has passed then ϕ_{Sept} will be removed from equation (18) i.e. ($\sum_{\forall j} \phi_j = \phi_{Oct} + \phi_{Nov} + \phi_{Dec}$). The following pro-active approaches will be combined with the baseline passive vaccination.

(a) **Random Vaccination**

For the random vaccination policy (RV), each day a number of individuals are selected at random and told about the upcoming or current flu clinics.

(b) **Nearest Neighbor**

The nearest neighbor approach (NN) (or acquaintance approach) is similar to the RV, in the way that each day a number of individuals are selected at random. However, the randomly selected individuals now recommends a "friend" for vaccination as well. [18]

(c) **Chain Vaccination**

The chain vaccination approach (CV) is similar to the NN; however, the policy consists of moving from one node to another with probability Δ and jumping to a random node with probability $1-\Delta$ [19,20]. The chain vaccination approach is sometimes referred to as a Page Rank process [19]. The CV approach would be considered a chain vaccination strategy, since recruitment runs along a chain of contacts until the chain is broken. That is, the following day public health would inform a contact of

the individual about the vaccination program with probability Δ; where another random individual would be contacted the following day with probability $1-\Delta$. Δ could represent the probability of complying to recommending a friend. We assumed a value of 0.50 for Δ.

(d) **Improved Nearest Neighbor**

The improved nearest neighbor (INN) approach is the same process as NN; however, now the random individual is asked to recommend a contact for vaccination who they view as "popular". This will allow for the higher degree individuals to more likely be recommended for vaccination. An approach similar to this was taken in Refs [20, 21]; the assumption allows for the individual to have some knowledge, but not complete knowledge, about the degree of their contacts. To implement such a process we use a cumulative distribution function approach, where each contact is assigned a weight corresponding to their degree size. The process consists of choosing a random number $0 < r < 1$ and finding contact j such that

$$r\sum_{n=1}^{k_i} k_n < \sum_{n=1}^{j} k_n \text{ where } j \leq k_i, \tag{22}$$

such that j is the smallest possible number to satisfy equation (22), k_n is the degree for i's n^{th} contact and k_i is the degree of i. This will allow for some error in the individual's incomplete knowledge of their contacts degree.

Quality Adjusted Life Years

To monetize the risk of infection and the risk associated with vaccination we used Quality Adjusted Life Years (QALYs). The cost of infection ($\overline{c_{inf}}$) is based upon the QALY penalties due to flu symptoms, plus the QALY penalty due to death, and can be expressed as

$$\begin{aligned}\overline{c_{inf}} &= \text{(prob. infection this year)} \times \text{(cost of} \\ &\quad \text{infection in QALY's)} \times (\$/\text{QALY}).\end{aligned} \tag{23}$$

The probability of infection this year corresponds to the incidence of the population, the cost of infection expressed in QALY's equals (QALY penalty from symptoms) + (probability of death)x(QALY penalty from death). The value for the QALY penalty from influenza symptoms is 4.3/365, where 4.3 is the Quality Adjusted Life Days penalty from influenza symptoms [22], and the QALY penalty from death is 15, where the probability of death is 0.00075 [22].

For the cost of experiencing a vaccine complication, we calibrated the value of $\gamma\times$(cost of vaccination in QALY's) such that it was the same magnitude as (prob. infection this year) $\times$ (cost of infection in QALY's). We express the cost of vaccination as

$$\begin{aligned}\overline{c_{vac}} &= \gamma \times \text{(cost of vaccination in QALY's)} \\ &\times \quad (\$/\text{QALY}),\end{aligned} \tag{24}$$

where γ is the probability of a vaccine complication occurring.

The value of $\overline{c_{inf}}$ was based upon utility penalties constructed from patient surveys [22]. The value of $\underline{c_{vac}}$ was based on published vaccine costs and held fixed for the calibration of the vaccine coverage. Since vaccine complications are rare,the value of $\overline{c_{vac}}$ was obtained by determining the cost of vaccine complications in QALYs such that the probability of complications (γ) times the cost of vaccine complications in QALYs was similar magnitude as incidence times the cost of influenza infection in QALYs.

Cost of Policy

We had to determine an estimated cost for the policies to determine whether the cost of incentives outweigh the cost saved from infection. The average cost of a single influenza infection is \$100.00, where the cost of a single vaccine is \$20.00 [23]. To determine the cost of incentives we used the percentage of incentives used and multiplied by the total number distributed and the monetary value of the incentive. Therefore, the total cost of a policy can be expressed as

$$\begin{aligned} \text{Cost} &= N \times (\$100.00\langle I(t)\rangle + \$20.00\langle V(t)\rangle) \\ &+ 122\bar{V}\Upsilon(\%\text{used}), \end{aligned} \tag{25}$$

where N is the population size, $\langle I(t)\rangle$ is the average annual incidence, $\langle V(t)\rangle$ is the average annual vaccine uptake, $\bar{V}$ is the number of individuals recruited each day and given an incentive, Υ is the monetary value of the incentive and %used is the percentage of incentives used each year.

Algorithm Summary

For each network we generated 400 simulations of 125 years each, discarding the transient dynamics of the first 100 years, with no vaccination occurring in the first year of each simulation. The following steps were taken to implement the behavior and vaccination process after the initial non-vaccination year. Prior to the vaccination season

1. Update the individual's perceived vaccine efficacy
2. Individual's proceed through the learning (imitation) process
   - i) Determine whether the individual imitates or not
   - ii) If they imitate randomly select a contact and individual from the network
   - iii) Assign the individual their new information using equations (19)-(21)
3. Individual determines both $\mathcal{P}_{\mathcal{V}}$ and $\mathcal{P}_{\mathcal{N}}$
4. Individual determines whether or not they are going to vaccinate this season
5. Then we determine when the Individual vaccinates

Afterwards, during the time period of the vaccination program, the following steps are implemented daily for the vaccination program

1. We randomly choose $\bar{V}$ individuals and recommend vaccination
2. If the selected individual decided not to vaccinate this season they repeat Step 2 to Step 5 above
   - i) If the selected individual had originally decided to vaccinate this season then do not repeat Step 2 to Step 5 above
3. If the individual vaccinates today
   - i) Determine whether or not the vaccine was effective
   - ii) Determine whether or not the individual experienced a complication
   - iii) If the individual experienced a complication set the individuals time since last vaccine complication to -1 day ($T_C = -1/365$)

The overall model with vaccination and infection follows the process below

1. Determine whether the individual looses vaccine immunity
2. Determine whether the individual looses natural immunity
3. Apply the process of determining whether the individual vaccinates this year and when
4. Start the year and vaccination program
5. Add one day to both T_I and T_C (i.e. $T_I = T_I + 1/365$)
6. If time corresponds to period of vaccination program
   i) Randomly select individuals to inform about vaccination
   ii) Apply the process of determining whether the randomly selected individuals vaccinate this year
   iii) Follow the process for an individual vaccinating today
7. If time corresponds to introducing an infected individual
   i) Randomly select a susceptible individual to be infected with influenza
8. Compute the individuals probability of becoming infected today
9. Determine if the individual becomes infected and determine if symptomatic
10. If the individual is infected and the flu is symptomatic
    i) Set the individuals time since last infection to -1 day ($T_I = -1/365$)
    ii) If the individual vaccinated set the perceived vaccine efficacy to $\underline{\varepsilon}$
11. Determine whether the individual leaves the infectious state or not
12. Infect the population with niILI
13. If individual is infected with ILI determine whether niILI is mistaken for influenza
14. If niILI is mistaken for influenza
    i) Set the individuals time since last infection to -1 day ($T_I = -1/365$)
    ii) If the individual vaccinated set the perceived vaccine efficacy to $\underline{\varepsilon}$
15. If end of year go to Step 1, otherwise go to Step 5 until the desired time is reached

References

1. Network Dynamics and Simulation Science Laboratory (2008). Synthetic data products for societal infrastructures and proto-populations: Data set 1.0. Virginia Polytechnic Institute and State University.
2. Network Dynamics and Simulation Science Laboratory (2008). Synthetic data products for societal infrastructures and proto-populations: Data set 2.0. Virginia Polytechnic Institute and State University.

3. Network Dynamics and Simulation Science Laboratory (2008). Synthetic data products for societal infrastructures and proto-populations: Data set 3.0. Virginia Polytechnic Institute and State University.

4. Mao L (2011) Agent-based simulation for weekend-extension strategies to mitigate influenza outbreaks. BMC Public Health 11.

5. Truscott J, Fraser C, Hinsley W, Cauchemez S, Donnelly C, et al. (2009) Quantifying the transmissibility of human influenza and its seasonal variation in temperate regions. PLoS Currents 1: RRN1125.

6. Keeling M, Rohani P (2008) Modeling Infectious Diseases in Humans and Animals, Princeton Press. p. 21.

7. Truscott J, Fraser C, Cauchemez S, Meeyai A, Hinsley W, et al. (2012) Essential epidemiological mechanisms underpinning the transmission dynamics of seasonal influenza. J R Soc Interface : 304-312.

8. Dushoff J, Plotkin J, Levin S, Earn D (2004) Dynamical resonance can account for seasonality of influenza epidemics. PNAS 101: 16915-16916.

9. CDC (2012). Influenza vaccination coverage: Fluvaxview:2010-11 influenza season. `http://www.cdc.gov/flu/professionals/vaccination/vaccinecoverage.htm`.

10. Bridges C, Thompson W, Meltzer M, Reeve G, Talamonti W, et al. (2000) Effectiveness and cost-benefit of influenza vaccination of healthy working adults: A randomized controlled trial. JAMA 284.

11. Couch R (1993) Advances in influenza virus vaccine research. Annals of the New York Academy of Sciences 685: 803-812.

12. CDC (2012). 2011-2012 influenza season: Disease activity. `http://www.cdc.gov/flu/about/season/flu-season-2011-2012.htm`.

13. CDC (2012). The flu season. `http://www.cdc.gov/flu/about/season/flu-season.htm`.

14. Lloyd-Smith J, Schreiber S, Kopp P, Getz W (2005) Superspreading and the effect of individual variation on disease emergence. Nature 438: 355-359.

15. Galvani A, May R (2005) Dimensions of superspreading. Nature 438: 293-295.

16. Funk S, Gilad E, Watkins C, Jansen V (2009) The spread of awareness and its impact on epidemic outbreaks. PNAS 106: 6872-6877.

17. Bansal S, Grenfell B, Meyers L (2007) When individual behaviour matters: homogeneous and network models in epidemiology. J R Soc Interface 4: 879-891.

18. Cohen R, Havlin S, ben Avraham D (2003) Efficient immunization strategies for computer networks and populations. Physical Review Letters 91: 247901.

19. Miller J, Hyman J (2007) Effective vaccination strategies for realistic social networks. Physica A 386: 780-785.

20. Holme P (2004) Efficient local strategies for vaccination and network attack. Europhysics Letters 68: 908-914.

21. Kim B, Yoon C, Han S, Jeong H (2002) Path finding strategies in scale-free networks. Physical Review E 65: 027103.

22. Turner D, Wailoo A, Cooper N, Sutton A, Abrams K, et al. (2006) The cost-effectiveness of influenza vaccination of healthy adults 50-64 years of age. Vaccine 24: 1035-1043.

23. Wood S, Nguyen V, Schmidt C (2000) Economic evaluations of influenza vaccination in healthy working-age adults employer and society perspective. Pharmacoeconomics 18: 173-183.
